# Antifungal Fluconazole Induces Aneuploidy, Sowing the Seeds of Its Own Failure

**DOI:** 10.1371/journal.pbio.1001816

**Published:** 2014-03-18

**Authors:** Richard Robinson

**Affiliations:** Freelance Science Writer, Sherborn, Massachusetts, United States of America

[Fig pbio-1001816-g001]A yeast infection is, at best, seriously annoying, causing itching and redness at the site of infection, usually the mouth, genitals, or nail beds. At worst, when the fungus spreads systemically, infection can be life-threatening. The antifungal arsenal is far smaller than the antibacterial one, comprising only a few classes of agents. Fluconazole, in the azole class, is the most widely used antifungal drug worldwide. Fluconazole is highly effective, but resistance is becoming increasingly common.

**Figure pbio-1001816-g001:**
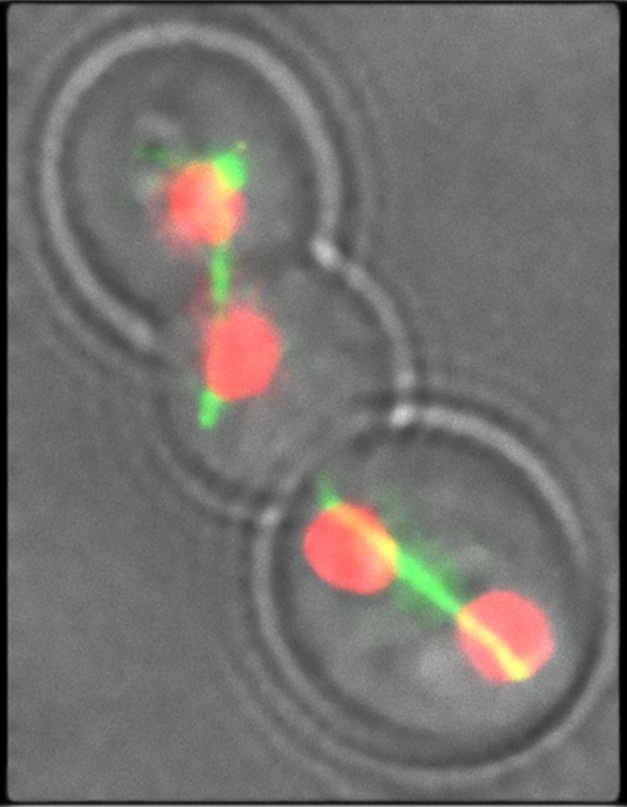
The antifungal drug fluconazole induces *Candida albicans* to form a novel cell type—a “trimera”—that contains two simultaneously dividing nuclei. Mitotic spindles (green) are shown segregating sister nucleoli (red). *Image by Benjamin D. Harrison*.

*Candida albicans* is one of the most common causes of yeast infections, and within the last decade, it has become clear that in drug-resistant strains of *C. albicans*, up to half of isolates exhibit aneuploidy—an abnormal number of chromosomes. Aneuploidy likely contributes to drug resistance by providing extra copies of drug-resistance genes. What hasn't been clear is whether that aneuploidy preexists in *C. albicans* populations, and is simply selected when non-aneuploid and non-resistant cells are killed, or whether aneuploidy is induced as a consequence of the treatment itself. In this issue of *PLOS Biology*, Benjamin Harrison, Judith Berman, and colleagues show that fluconazole and other related azole antifungals induce aneuploidy by disrupting the normal synchronization of chromosomal and cytoplasmic division.

The authors began by measuring the DNA content of yeast cells as a function of time of exposure to fluconazole. The quantity of DNA in untreated cells was standard—either 2N (corresponding to the normal diploid chromosome number) or 4N (after DNA replication but before cell division). But after 8 hours of drug exposure, up to a third of cells contained 8N, and longer exposures increased it even beyond that.

Yeast multiply by budding and cytokinesis, a process in which the cytoplasm of a cell is divided in two. Replication and mitosis—the duplication and redistribution of chromosomes—are normally coordinated with cytokinesis to ensure equal division of chromosomes between mother and daughter cells. But microscopy revealed that many fluconazole-treated cells failed to pinch off after budding, creating multimeric chains. Most common was the “trimera,” with mother, daughter, and granddaughter linked together. Some of these cells had multiple nuclei.

To better understand the consequences of trimera formation, the authors compared the coordination of cell cycle events between treated and untreated cells. They found that fluconazole treatment was associated with a delay in bud formation, but without a coordinated delay in either DNA replication or duplication of the spindle pole body, which organizes chromosome separation. The two nuclei often successfully underwent a second mitosis coincident with budding of a single granddaughter, leaving four nuclei, four spindle pole bodies, and one cytoplasm distributed among three cell compartments. In some cases one of the compartments retained two separate nuclei, while in others a pair of nuclei collapsed to form a single tetraploid nucleus.

Normally, a daughter cell inherits only a single spindle pole body. But in trimeras, the compartment with an extra nucleus also had an extra spindle. Therefore, after duplication, four, not two, spindle poles competed to separate either a diploid or tetraploid set of chromosomes. Havoc, and aneuploidy, ensued.

To test the *in vivo* relevance of these observations, the authors observed *C. albicans* growing in the ears of mice, with or without fluconazole. Yeast in untreated mice grew normally, with no signs of trimeras; they also produced infectious hyphae. In treated mice, yeast growth was inhibited, but trimera formed in about 20% of cells.

Fluconazole inhibits the growth of fungal membranes without killing them, which likely explains the results seen here: treatment disrupted the normal coordination of membrane and nuclear events, leaving chromosomes to separate without a new cell to separate into, forming tetraploids. Treatment means that some tetraploids will become aneuploid, and some aneuploids will be overloaded with drug-resistance genes.

The discovery of this phenomenon is troubling, since it means that treatment sows the seeds of its own failure. Since the yeast are inhibited but not killed, they remain in place even after successful treatment, and longer treatment doesn't eradicate the resistant cells. But understanding the origin of that resistance may allow the design of drugs that can either overcome it or exploit a pathway that avoids it.

**Harrison BD, Hashemi J, Bibi M, Pulver R, Bavli D, et al. (2014) A Tetraploid Intermediate Precedes Aneuploid Formation in Yeasts Exposed to Fluconazole.** doi:10.1371/journal.pbio.1001815

